# PathEx: a novel multi factors based datasets selector web tool

**DOI:** 10.1186/1471-2105-11-528

**Published:** 2010-10-22

**Authors:** Eric Bareke, Michael Pierre, Anthoula Gaigneaux, Bertrand De Meulder, Sophie Depiereux, Naji Habra, Eric Depiereux

**Affiliations:** 1 Molecular Biology Research Unit (URBM), University of Namur - FUNDP, Namur, Belgium; 2 Research Center in Information Systems Engineering (PReCISE), University of Namur - FUNDP, Namur, Belgium

## Abstract

**Background:**

Microarray experiments have become very popular in life science research. However, if such experiments are only considered independently, the possibilities for analysis and interpretation of many life science phenomena are reduced. The accumulation of publicly available data provides biomedical researchers with a valuable opportunity to either discover new phenomena or improve the interpretation and validation of other phenomena that partially understood or well known. This can only be achieved by intelligently exploiting this rich mine of information.

**Description:**

Considering that technologies like microarrays remain prohibitively expensive for researchers with limited means to order their own experimental chips, it would be beneficial to re-use previously published microarray data. For certain researchers interested in finding gene groups (requiring many replicates), there is a great need for tools to help them to select appropriate datasets for analysis. These tools may be effective, if and only if, they are able to re-use previously deposited experiments or to create new experiments not initially envisioned by the depositors. However, the generation of new experiments requires that all published microarray data be completely annotated, which is not currently the case. Thus, we propose the PathEx approach.

**Conclusion:**

This paper presents PathEx, a human-focused web solution built around a two-component system: one database component, enriched with relevant biological information (expression array, omics data, literature) from different sources, and another component comprising sophisticated web interfaces that allow users to perform complex dataset building queries on the contents integrated into the PathEx database.

## Background

Although there has been a trend whereby many researchers widely use microarray technologies, less is done computationally to interpret and validate biological hypotheses formulated from inherent investigation results. Continued microarray data deposit and revision of genome annotations are important to supplement previously submitted microarray metadata. While the advent of microarray technologies and an increasing number of analysis methods present an opportunity to better understand life mechanisms, exploitation of microarray data and the choice of analysis methods remain challenges. The idea behind the development of PathEx originates from a benchmarking study we conducted comparing microarray statistical analysis methods [1]. During the study, it was found that some methods focusing on finding gene groups might require many replicates. For a researcher considering conducting a microarray analysis, one consideration should be taken into account: the dataset of interest.

At this level, the challenges include (a) how to effectively and more easily obtain a dataset with the number of replicates necessary for the analysis method chosen and (b) how to select a dataset for a specific purpose (e.g., study of a specific pathology and study of a specific drug response) to increase the statistical power of the analysis method. One way to effectively meet these needs would be to consider re-using previously deposited microarray data from the same or different studies (with different biological hypotheses) without necessarily conducting new experiments.

We propose here a novel web tool that combines information from microarray data, the literature and omics technologies. Its main objective is to allow for instantaneous selection and generation of datasets of interest by drawing relevant samples files from major publicly available microarray repositories and using simple but biologically meaningful keywords to query the underlying database. PathEx provides biologists (with no or limited pre-knowledge of the structure and organization of the microarray data) with an intuitive web interface to generate datasets for validation of existing studies, discovery of new phenomena or complementation of hypotheses regarding phenomena only partially understood.

Many researchers must often manually retrieve or use certain tools available to retrieve microarray data from public repositories. However, such tools are most often limited to pre-knowledge of the structures and formats of the deposited microarray data.

Several tools proposed are mainly either retrieval tools (Microarray Retriever (MaRe) [2]) or full integrated but manufacturer-oriented analysis tools (combining retrieval and analysis tools: EzArray [3] and SiPaGene [4]). However, none have the enhanced ability to allow researchers to automatically select data of interest by focusing on certain biological factors that were not necessarily those provided in the microarray metadata.

Unlike existing tools, the power of PathEx is its fast processing capability made possible through local storage of all of the data (to avoid the sequential downloading policies and bandwidth limitation associated with most microarray repositories). PathEx also remains unique in that it acts as a point of integration of fully re-organized information from public sources. Furthermore, PathEx is not bound to any microarray manufacturer or type. This allows for the datasets selected by PathEx to be analyzed by any platform associated analysis method.

## Construction and Content

### Rationale for PathEx

As PathEx does not aim to be yet another microarray retrieval tool and the main goal was to develop a novel concept to offer less exploited opportunities for the analysis of deposited microarray data. Deposited microarray data comes with description files (though these files are sometimes incomplete). These metadata files do however contain some key information that can be used to link the microarray data to other biologically related information. We propose here a system that uses this identification metadata to link microarray data to other biological concepts such as *Genes*, *Proteins*, *Metabolic Pathways *and *the Literature*. By further characterizing previously deposited microarray data; we provide researchers with new opportunities to select interesting datasets by simply using meaningful biological criteria to query the underlying PathEx database.

### Implementation of PathEx

To implement PathEx, we used the popular LAMP bundle, where LAMP stands for Linux operating system http://www.linux.org/; Apache web server http://www.apache.org/, MySQL relational database management system http://www.mysql.com/, PHP http://php.net/ and Perl http://www.perl.org/. A set of new web technologies such as Asynchronous JavaScript And XML (AJAX, http://www.w3schools.com/ajax/default.asp), JavaScript Query (JQuery, http://jquery.com/), MooTools http://mootools.net/ and JavaScript Object Notation (JSON, http://www.json.org/) was mainly used to increase the system's interactivity, functionality and versatility.

### Data Management and Sources for PathEx

The purpose of PathEx is to allow for custom selection of microarray datasets by completing microarray annotation with biological information from different and heterogeneous sources. While microarray data is automatically drawn from the National Center for Biotechnology Gene Expression Omnibus (NCBI GEO) [5] and European Bioinformatics Institute Array Express (EBI AE) [6-9], the biological information used to further characterize that data is mainly taken from major omics databases/databanks. The idea is to establish a link between microarray metadata and other widely used cross-reference entries, opening up new complex query possibilities. The database component of PathEx currently includes gene information from the NCBI (through Entrez Gene [10] system) and Kyoto Encyclopedia of Gene and Genomes (KEGG) [11-13], ENSEMBL [14-20], H-InvDB [21,22], Vertebrate Genome Annotation (Vega) [23,24], protein information from UniProt/Swiss-Prot [25] and ENSEMBL and metabolic pathway information from KEGG Pathways.

However, one of the challenges we faced when dealing with publicly available biological data was the lack of appropriate tools to organize that data and overcome critical issues such as different file formats, ontologies, structures and accessibilities, lack of information about the contents provided (e.g. KEGG) and incomplete annotation.

Although some sources have opened up their contents to the public by different means such as application programming interfaces (API) and other programmatic tools, the issues mentioned above hamper automated retrieval processes.

To overcome these constraints and provide researchers with an automated criteria-driven dataset selector, we developed a set of complex tools to deal with these issues through step-by-step conversion of the contents into open formats and collection of the relevant data to be integrated into the database. The power of these tools relies on how they effectively handle different constraints (e.g. data formats, data structures, accessibility) by independently importing locally and converting all required data to populate the PathEx database.

To ensure the reliability and quality of the data collected, a team of biology experts scrutinized and cross-checked it wherever necessary.

### Architecture and Design of PathEx

**Figure 1:**
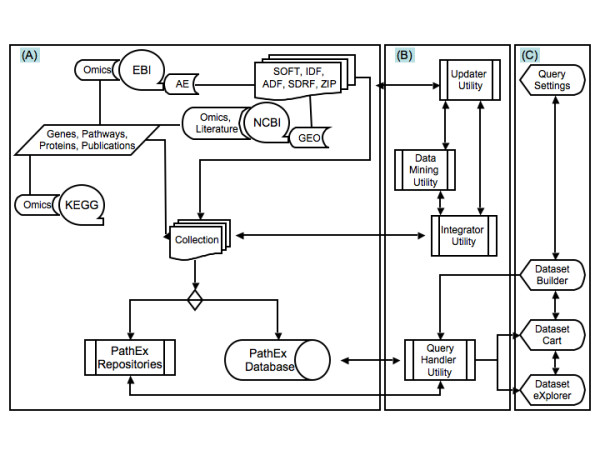
**PathEx system architecture (PathEx main process communications)**.

The PathEx architecture is divided into three main components (Figure 1): The Processing Logic, The Contents Logic and The Navigator Logic. The Processing Logic has four interdependent utilities (Data Mining Utility, Integration Utility, Query Handler Utility and Updater Utility), The Contents Logic has two storage approaches (Database and Files Repository) and The Navigator Logic has several interfaces (Query Settings, Dataset Builder, Dataset Cart and Global Datasets Manager).

#### The PathEx Processing Logic

The PathEx Processing Logic is responsible for:

1. Federation of:

a. Basic microarray data (platforms, experiments and samples) and associated sample raw data from GEO Simple Omnibus Format in Text (SOFT, http://www.ncbi.nlm.nih.gov/geo/info/soft2.html#SOFTformat) files and AE MicroArray and Gene Expression tab (MAGE-TAB) [26]) files,

b. Additional reviewed microarray metadata, not primarily envisioned by the experiment owners (biological tags: sex, tissue, organ) and

c. Biological information (genes, proteins, metabolic pathways and literature information),

2. Remote change tracking and updating whenever required,

3. PathEx user and query management and

4. PathEx database integration.

**Figure 2:**
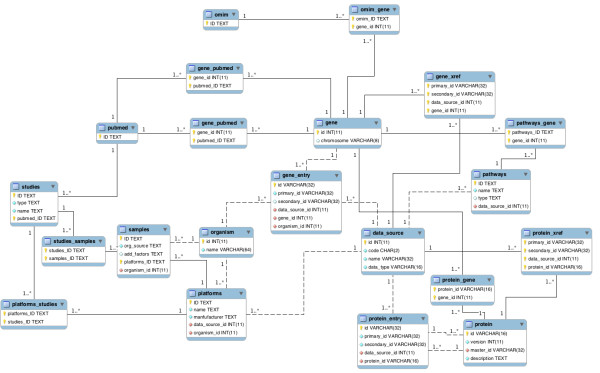
**Simplified PathEx Database Enhanced Entity Relationship Data Model (EER DM)**.

As one of the back end components of PathEx, The Data Mining Utility provides a set of algorithms to extract, parse, organize, correlate and convert relevant information: Microarray data (e.g. .CEL files) and metadata, Genes, Proteins, Pathways and Literature information. The Integration Utility manages a relational database (Figure 2) component by loading into and updating it with appropriate structured data. The Query Handler Utility that negotiates the dataset build by checking submitted selection criteria and filters and invoking necessary sample files to build a dataset handles all user queries. PathEx, through the Updater Utility, provides a schema-evolution service that is valuable because the ongoing revision of biological data and the complexity of bioinformatics schemas imply that they are always evolving.

#### The PathEx Contents Logic

This component manages the PathEx data storage system: (a) the File Repositories of microarray data files: SOFT files (from GEO), MAGE-TAB files (from AE) and different biological source files used to enrich microarray characterization and (b) the Database containing structured and related microarray metadata and annotational information. GEO SOFT files contain data tables and the accompanying descriptive information for multiple, concatenated Platforms, Samples, and/or Series records.

The integrated AE MAGE-TAB files consist of four different types of files: (a) A "raw" zip archive contains the raw data files, i.e. the files produced by the microarray image analysis software, such as CEL files for Affymetrix GeneChip, (b) The Array Design Format (ADF) tab-delimited file describes the design of an array, (c) The Investigation Description Format (IDF) tab-delimited file contains top-level information about the experiment including the title, description, submitter contact details and protocols and (d) The Sample and Data Relationship Format (SDRF) tab-delimited file containing the relationships between the samples and arrays, as well as sample properties and experimental factors, as provided by the data submitter.

#### The Navigator Logic

This component comprises a set of intuitive, interactive and easy-to-use web interfaces. They provide users with features to customize and select a dataset simply by specifying criteria not initially envisioned by those who deposited the expression array data.

## Utility and Discussion

To conduct a routine microarray study analysis, we need (a) a dataset of interest, (b) an appropriate analysis method and (c) a means to evaluate, interpret and validate the results obtained. Currently, benchmarking studies have often emphasized the importance of selection of the analysis methods. This agrees with our recent benchmarking analysis, where we showed that the choice of appropriate analysis methods is crucial for the accuracy of the expected results. Recently, a re-analysis conducted on Golden Spike data by Pearson [27] outlined the characteristics of an ideal dataset: (a) a realistic spike-in concentration, (b) a mixture of up- and down-regulated genes, (c) unrelated fold change and intensity and (d) *a large number of arrays*. Based on these criteria, we believe that custom selection of a dataset to analyze is crucial.

As the principal objective of a microarray analysis is to reduce variability, we should consider unexploited ways to do this, particularly in light of the outcome of several studies [28,29] that postulated a complex relationship between variability and expression level. We think that, without minimizing other sources, variability can be reduced by intelligently selecting a focused dataset (e.g. dataset related to a specific pathway, pathology, organ and other factors)

However, as there are no existing tools to automatically select such a dataset, PathEx constitutes an important tool in this context.

**Figure 3:**
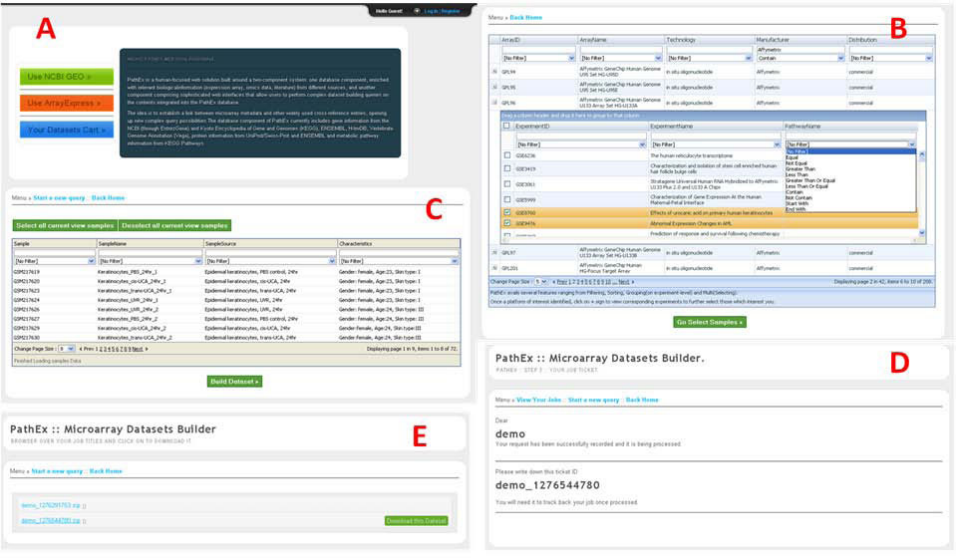
**Snapshots of some PathEx navigational interfaces**. The above interfaces present: (A) the entrance page after login, (B) the available features such as Multi Selection and Filtering, (C) the dataset build ticketing system and (D) the dataset explorer cart.

With its enriched content and advanced selection features, PathEx provides simple and easy-to-use interfaces (Figure 3) to help users avoid the burden of thinking about complex queries. It combines flexibility, fast processing, accuracy and an easy-to-understand search system using biological tag criteria.

With its multiple level consecutive query interfaces, PathEx maximizes the user searching process and keeps users informed of each query task filter output at each level of dataset selection within an interactive grid. A user is provided with a specific area and interfaces according to settings chosen on the entrance page. PathEx provides three-level selection interfaces, related consecutively on the organizational levels of the microarray data (platforms, studies and samples). Besides a search area, coupled with a set of filters ("equals", "contain", "does not contain" and others) at each level to allow for criteria-driven selection of datasets, there are advanced features to ease selection such as grouping, sorting and multiselecting.

Through the navigational settings, the user specifies the kind of keywords to query PathEx, to allow PathEx to display a customized interface. This approach was chosen to ease dataset selection and present clear interfaces. Many keyword types can be used to query PathEx (e.g. Accessions: gene IDs, gene symbols, protein IDs, OMIM number, and PubMed IDs; Factors: Metabolic pathway names, pathology names, tissues, organ and experiment types).

For each dataset selection request, a user is given a building ticket to trace the job process and download it when finished. The outcome is a compressed file containing all samples files related to the criteria submitted.

There are two ways of retrieving the datasets generated. Any user may retrieve its own datasets through the job cart, as it is name-driven. To evaluate the performance of PathEx, we tested it by selecting a customized dataset related to *"lung cancer"*from *"all""GEO"*experiments of the type *"Affymetrix"*GeneChip *"HG-U133A"*. By submitting the five highlighted search keys to PathEx and applying appropriate filters, we ended up, in less than 30 seconds, with a dataset of 108 samples.

It is important to note that generated datasets should be analyzed carefully given the known variability due to microarray design and phenotypic differences between studies. However grouping the datasets properly according to some specific characteristics can decrease the variability of a meta-analysis.

### Case Study

In our recently published work[30], we tried to evaluate the effectiveness of PathEx. We used it to try to find genes involved in the metastasis of cancer cells induced by hypoxia. Though many advances have been made in this field, all of the mechanisms involved are still not well understood. It is known that the expression of specific genes is modified in primary tumor cells to detach, migrate and invade surrounding tissues. But the integration of all of the associated data is a problem.

In the first phase of our meta-analysis, we used PathEx to select datasets about metastasis and/or hypoxia. Out of the 24 retrieved datasets, 17 were retained for further analysis (Table 1). As some of the selected datasets were not available in GEO or AE, we contacted the original authors to obtain them.

**Table 1:** Datasets used in the case study differential analysis.

Experiment/Study Accession numbers	Platform	Source	Availability	Experimental conditions
E-GEOD-1323	HG-U133A	AE	Available	3 human colorectal cancer derived from a primary tumor VS. 3 corresponding lymph node metastases

E-GEOD-2280	HG-U133A	AE	Available	8 squamous cell carcinoma of the oral cavity VS. 19 corresponding lymph node metastases

E-MEXP-44	HG-U95Av2	AE	Available	15 head and neck squamous cell carcinoma VS. 3 corresponding lymph node metastases

	HG-UgeneFL			12 head and neck squamous cell carcinoma VS. 11 corresponding lymph node metastases

GSE1056	HG-U95Av2	GEO	Not available	2 human hepatocellular carcinoma under hypoxia for 2 hours VS. 2 control human hepatocellular carcinoma 2 human hepatocellular carcinoma under hypoxia for 24 hours VS. 2 control human hepatocellular carcinoma

GSE2280	HG-U133A	GEO	Available	22 squamous cell carcinoma of the oral cavity VS. 5 corresponding lymph node metastases

GSE2603	HG-U133A	GEO	Available	100 primary breast cancer VS. 21 lung metastases

GSE3325	HG-U133Plus2.0	GEO	Available	7 primary prostate cancer VS. 6 metastases

GSE4086	HG-U133Plus2.0	GEO	Available	2 human Burkitt's lymphoma under hypoxia VS. 2 control human Burkitt's lymphoma

GSE468	HC-G110	GEO	Available	13 primary medulloblastomas VS. 10 metastatic medulloblastomas

GSE4840	HG-U133A	GEO	Not available	3 samples from normal melanocyte culture VS. 12 samples from culture of cutaneous metastasis of melanoma
				
	HG-U133B			3 samples from normal melanocyte culture VS. 12 samples from culture of cutaneous metastasis of melanoma

GSE4843	HG-U133Plus2.0	GEO	Not available	45 samples from culture of cutaneous melanoma metastasis

GSE6369	HG-U133Plus2.0	GEO	Available	1 primary prostate carcinoma VS. 1 metastatic prostate carcinoma

GSE6919	HG-U95Av2	GEO	Available	65 primary prostate tumors VS. 25 metastatic prostate tumors
				
	HG-U95B			66 primary prostate tumors VS. 25 metastatic prostate tumors
				
	HG-U95C			65 primary prostate tumors VS. 25 metastatic prostate tumors

GSE7929	HG-U133A	GEO	Available	11 poorly metastatic melanoma VS. 21 highly metastatic melanoma

GSE7930	HG-U133A	GEO	Available	3 poorly metastatic prostate tumors VS. 3 highly metastatic prostate tumors

GSE7956	HG-U133A	GEO	Available	10 poorly metastatic melanoma VS. 29 highly metastatic melanoma

GSE8401	HG-U133A	GEO	Available	31 primary melanoma VS. 52 melanoma metastasis

The above table shows the list of datasets drawn by the PathEx system automatically.

In the second phase, we again used PathEx to generate 14 customized meta-datasets from the 17 original datasets (Table 2).

**Table 2:** Datasets used in the case study meta-analysis

Meta-dataset Name	Experimental conditions	GeneChip models	Datasets
Meta-dataset 1	Primary tumor, normal tissue, poorly metastatic tissue VS. metastasis, highly metastatic tissue	HG-U133A	E-GEOD-1323, E-GEOD-2280, GSE2280, GSE2603, GSE4840 (HG-U133A), GSE7929, GSE7930, GSE7956, GSE8401

Meta-dataset 2	Primary tumor, poorly metastatic tissue VS. metastasis, highly metastatic tissue	HG-U133A	E-GEOD-1323, E-GEOD-2280, GSE2280, GSE2603, GSE7929, GSE7930, GSE7956, GSE8401

Meta-dataset 3	Primary tumor, normal tissue VS. metastasis	HG-U133A	E-GEOD-1323, E-GEOD-2280, GSE2280, GSE2603, GSE4840 (HG-U133A), GSE7929, GSE7956, GSE8401

Meta-dataset 4	Primary tumor VS. metastasis	HG-U133A	E-GEOD-1323, E-GEOD-2280, GSE2280, GSE2603, GSE4840 (HG-U133A), GSE7929, GSE7956, GSE8401

Meta-dataset 5	Primary tumor VS. metastasis	HG-U133A	E-GEOD-1323, E-GEOD-2280, GSE2280, GSE2603, GSE7929, GSE7956, GSE8401

Meta-dataset 6	Squamous cell carcinoma of the oral cavity VS. corresponding lymph node metastases	HG-U133A	E-GEOD-2280, GSE2280

Meta-dataset 7	Normal melanocyte culture, poorly metastatic melanoma, primary melanoma VS. culture of cutaneous metastasis of melanoma, highly metastatic melanoma, melanoma metastasis	HG-U133A	GSE4840 (HG-U133A), GSE7929, GSE7956, GSE8401

Meta-dataset 8	Poorly metastatic melanoma, primary melanoma VS. culture of cutaneous metastasis of melanoma, highly metastatic melanoma, melanoma metastasis	HG-U133A	GSE4840 (HG-U133A), GSE7929, GSE7956, GSE8401

Meta-dataset 9	Poorly metastatic melanoma, primary melanoma VS. highly metastatic melanoma, melanoma metastasis	HG-U133A	GSE7929, GSE7956, GSE8401

Meta-dataset 10	Primary tumor VS. metastasis	HG-U95Av2	E-MEXP-44 (HG-U95Av2), GSE6919 (HG-U95Av2)

Meta-dataset 11	Hypoxia VS. normoxia	HG-U95Av2	GSE1056

Meta-dataset 12	Primary tumor, normoxia VS. metastasis, hypoxia	HG-U133Plus2.0	GSE3325, GSE4086, GSE4843, GSE6369

Meta-dataset 13	Primary tumor VS. metastasis	HG-U133Plus2.0	GSE3325, GSE4843, GSE6369

Meta-dataset 14	Primary prostate cancer VS. metastases	HG-U133Plus2.0	GSE3325, GSE6369

Using PathEx features, which allowed combination of samples from different experiment, we automatically created the above datasets.

**Figure 4:**
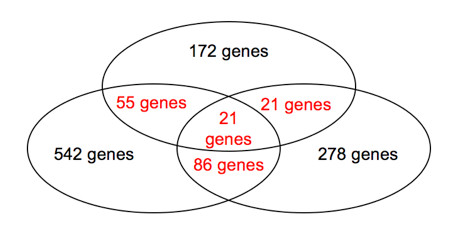
**Venn's diagram of interesting differentially expressed genes as revealed by the case study**.

After analysis, our study results were combined, highlighting 183 genes of interest (Figure 4). Out of these genes, 99 are already known in the literature to be involved in cancer, among which 39 in metastasis, while 21 are related to the response to hypoxia. The other genes of interest found by our methodology are now under investigation to determine their role in hypoxia-induced metastasis.

### Perspectives for PathEx

PathEx is currently a human-oriented web tool. It is likely to be extended to other species in the future. Currently indexed biological information such as pathways are limited to one source (KEGG), we are considering integrating other sources such as Wiki Pathways [31] for pathway information, expanding the query options given to users in the event of selective sources due to licensing issues linked to the information owners.

**Figure 5:**
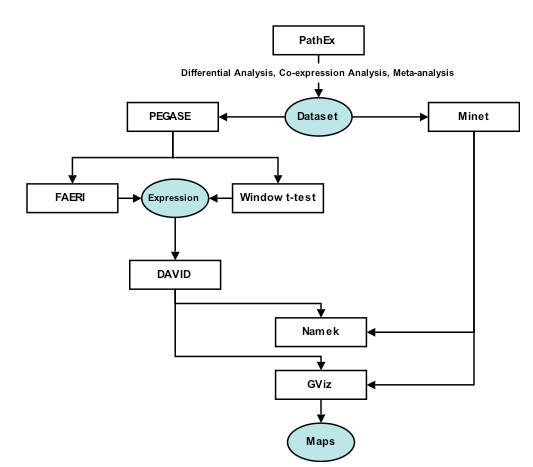
**Future planned PathEx development components**. The new integrated analysis tool will combine PathEx, proven analysis tools such Minet and DAVID and locally developed tools (PEGASE, FAERI, Window t-test, Namek and GViz).

As PathEx is not bound to any analysis method, we are currently developing a fully-automated and integrated Affymetrix web analysis tool to combine PathEx with analysis methods developed by us and proven to be efficient: the Window t-test and PHOENIX [32], interesting tools such as DAVID [33] and tools developed in-house (GViz and Namek). Besides the reasons stated above, additional factors such as recent redefinition approaches of Affymetrix Chip Definition Files (CDF) [34-36] and a large number of powerful analysis methods published enforce the utility of PathEx. We strongly believe that it will help researchers to automate their dataset selection. The choice will be up to them whether to do a single gene/gene group differential or co-expression analysis or a meta-analysis (Figure 5).

## Conclusions

Publicly available microarray data are rich mines of information. Fully exploiting them may help to understand life mechanisms. However, effective exploitation of this information requires us to further characterize them by crossing their metadata with other biological information.

We present here a database coupled with a web interface that, by intelligently organizing information from different biological sources, will allow researchers to select relevant datasets (mandatory initial step of any routine microarray analysis). We believe this will help to discover, interpret, validate and further develop biological hypotheses without the need to conduct new experiments.

## Availability and Requirements

PathEx is freely accessible for non-commercial users from http://urbm-cluster.urbm.fundp.ac.be/webapps/pathex/

Login parameters for testing:

Username: **demo**

Password: **138.48**

## Authors' contributions

EB designed and coded the major part of PathEx, drafted the manuscript and wrote the final version of the manuscript. ED, as the principal project initiator, supervised the project development at all levels, reviewed and approved the last version of the manuscript. NH, as co-director of the project, intervened during technical specification, provided advice on technical choices made, reviewed and approved the last version of the project. AG, BdM, and SD verified the database content quality, tested it and gave comments on the manuscript. MP tested the PathEx database and used it to generate the data used in the article published in BMC Cancer. All authors read and approved the final manuscript.
