# Correction: PathEx: A novel multi factors based datasets selector web tool

**DOI:** 10.1186/1471-2105-11-541

**Published:** 2010-11-02

**Authors:** Eric Bareke, Michael Pierre, Anthoula Gaigneaux, Bertrand De Meulder, Sophie Depiereux, Fabrice Berger, Naji Habra, Eric Depiereux

**Affiliations:** 1 Molecular Biology Research Unit (URBM), University of Namur - FUNDP, Namur, Belgium; 2 Research Center in Information Systems Engineering (PReCISE), University of Namur - FUNDP, Namur, Belgium

## Correction

During the proofing stage of our article [1] we made an error by forgetting to put on the list of authors a biology expert Fabrice Beger who cross-checked the reliability and the quality of our database, tested it and gave comments on the manuscript. We apologize for any inconvenience.
